# Descending necrotizing mediastinitis caused by *Streptococcus constellatus* in an immunocompetent patient: case report and review of the literature

**DOI:** 10.1186/s12890-020-1068-3

**Published:** 2020-02-17

**Authors:** Rui-hai Ye, Jun-chao Yang, Hui-hua Hong, Yu-ping Mao, Yuan-hong Zhu, Yu Cao, Zhen Wang

**Affiliations:** 1 Department of Respiratory Medicine, Zhejiang Province Hospital of Traditional Chinese Medical, Hangzhou, China; 2 Department of Laboratory, Zhejiang Province Traditional Chinese Medical Hospital, Hangzhou, China

**Keywords:** Descending necrotizing mediastinitis, *Streptococcus constellatus*, EBUS-TBNA, Calcified mediastinal lymph node

## Abstract

Descending necrotizing mediastinitis is a severe infection of the mediastinum. This syndrome manifests as fever and chest pain following cough and sputum production. A 49-year-old woman presented with fever and a 14-day history of pneumonia. CT showed mediastinal abscesses with a giant calcified mediastinal lymph node (21 × 18 mm) and pneumonia. Bronchoscopy by EBUS-TBNA under general anesthesia was performed. The pathogen found in the puncture culture was *Streptococcus constellatus*, and antibiotics (mezlocillin/sulbactam 3.375 IVGTT q8h) was administered. A proximal right main bronchial neoplasm, suspected lung cancer, was found and conformed to inflammatory granuloma. A total of 22 months post-discharge the patient was clinically stable. We also conducted a review of the literature for all *Streptococcus constellatus* descending necrotizing mediastinitis infections between 2011 and 2017.

## Background

Descending necrotizing mediastinitis (DNM) is an uncommon disease when the widespread antibiotics are used today, but accompanied by life threatening complication of infection in the oropharyngeal region that descends to the mediastinum through the connecting deep and superficial cervical fascial planes. The mortality rate of DNM is about 41%. It is approximately triples the risk of septic shock [1–3]. DNM is even more rarely caused by *Streptococcus constellatus*, a microorganism usually found in the normal flora of the human body [4].

## Case report

**Fig. 1:**
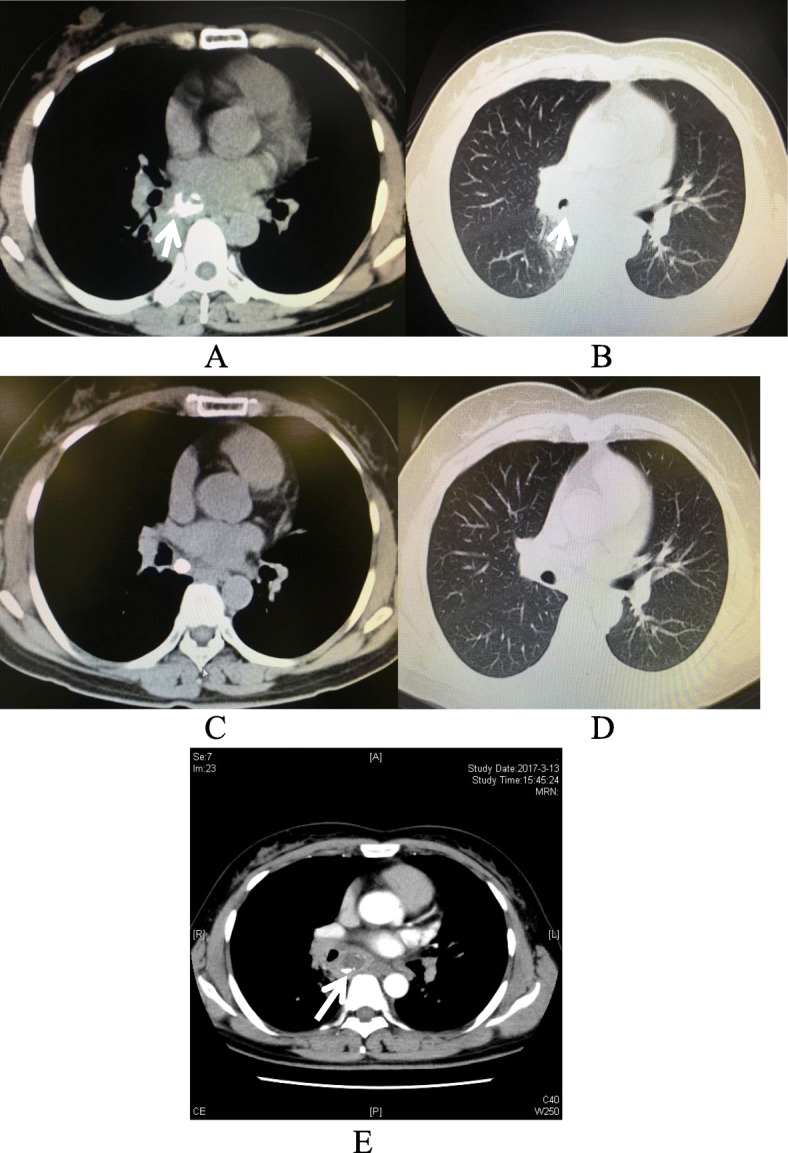
**a** Computed tomography of the mediastinum showing significantly enhanced signals for mediastinal abscesses with a giant calcified mediastinal lymph node (21 × 18 mm) (arrow). **b** Chest computed tomography showing a proximal right main bronchial neoplasm (5*4 mm) (arrow) and pneumonia in the right lower lobe. **c** Computed tomography of the mediastinum 22 months post-discharge showing significantly absorbed mediastinal abscesses with a slimmer calcified mediastinal lymph node (12 × 9 mm). **d** Chest computed tomography 22 months post-discharge showing complete resolution of pneumonia. **e** Chest Enhanceed Computed tomography of the mediastinum showing significantly low density for mediastinal abscesses with calcification (arrow)

**Fig. 2:**
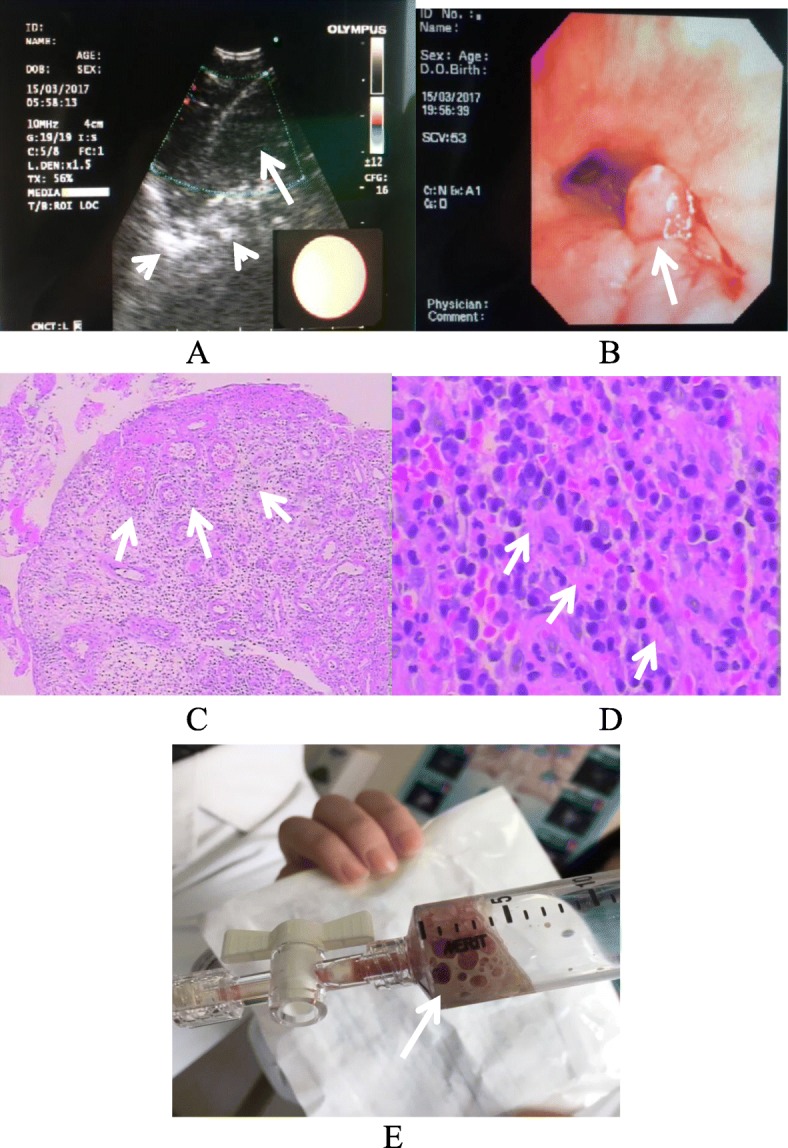
**a** EBUS-TBNA of the mediastinum showing hypodense loculated fluid (long arrow) and mediastinal lymph node calcification (short arrows). **b** Bronchoscopy of right principal bronchus showing a neoplasm (5 × 4 mm) (long arrow) with swelled and hyperemic mucosa. **c** Hematoxylin and eosin staining (× 100) showing pronounced necrosis and erosion of the bronchial mucosa, with inflammatory granuloma surrounding the bronchial mucosal tissue (arrows). **d** Hematoxylin and eosin staining (× 400) showing the bronchial mucosal tissue (arrows) surrounding large amounts of lymphocytes, plasmacytes and neutrophils. **e** EBUS-TBNA of the mediastinum revealing a foam-like purulent secretion of about 5 ml(arrow)

A 49-year-old female with a history of dental caries seeked medical advice to the outpatient Respiratory clinic with progressive fever. Her temperature fluctuated from 37.6 to 38.9 °C, following chest pain and productive cough for 14 days. There was a lot of white-yellow bubble-containing sputum. The patient denied rigors, night sweats, or exposure to sick contacts. However, she had a history of tuberculosis which was regularly cured since 30-years ago. On admission, the patient respiratory frequency is 16 breaths per minute and oxygen saturation is 98% with indoor quiet state. Her body temperature was 38.3 °C and blood pressure (BP) was 131/70 mmHg. There was decreased breath sound on the lower right zone of the chest by physical examination. There were no obvious rales or wheezes and no jugular vein distension either. Cardiac and abdominal exams were normal too. White blood count (WBC) was 6.9 × 10^9^/L (normal range, 3.5–9.5 × 10^9^/L). C-reactive protein (CRP) levels were 94 mg/L (normal range, 1–8 mg/L) with a erythrocyte sedimentation rate (ESR) of 66 mm/h (normal range, 0–20 mm/h). Antinuclear antibody (ANA) was positive 1:320(< 1:100). Vasculitis ANCA and human immunodeficiency virus (HIV) test were negative. Contrast-enhanced CT showed mediastinal abscesses with a giant calcified mediastinal lymph node (21 × 18 mm) (Fig. 1a, e) and pneumonia in the right lower lobe (Fig. 1b). Tumor markers、Electrocardiogram (ECG), pulmonary function, abdominal and retroperitoneum ultrasound were no abnormal changes. The empiric antibiotic treatment was started on intravenous moxifloxacin 0.4 ivgtt qd and cefoxitin2.0 ivgtt bid. Blood cultures (two sets) showed no special bacterial growth and sputum acid-fast bacilli (AFB; three sets) were negative either. For all that, the patient showed persistent fever (37.6–38.9) and chest pain following cough with yellow and white frothy sputum. Planed bronchoscopy of EBUS-TBNA under general anesthesia was performed (Fig. 2a), and turbid yellowish fluid was drawn (Fig. 2e). Subsequently laboratory analysis of the bronchoalveolar lavage fluid (BALF) showed a large number of pyogenic cells with left shift of nucleus. A proximal right main bronchial neoplasm(5*4 mm) (Fig. 2b) was unexpected found and the distal bronchus was unobstructed, also there was no change of atelectasis or obstructive pneumonia. It was conformed to inflammatory granuloma (Fig. 2c, d). This benign lesion was treated with bronchoscopic biopsy forceps and never cured with an ablative endoscopic procedure. It was disappeared in the following reexamination. Calcified lymph nodes in mediastinum were deduced to previous tuberculosis infection and it was eliminated the recurrent active infection of tuberculosis by laboratory examination. The endobronchial Ultrasound-Guided Transbronchial showed a circle low density shadow in mediastinum. EBUS-TBNA was done, whose model of puncture needle was Olympus 4022 with diameter of 22G and the max-length of 40 mm and suction foam-like purulent secretion of about 5 ml with 20cmH_2_O negative pressure. The operation lasted about half an hour. Subsequently culture of mediastinal abscess fluid yielded *Streptococcus constellatus,* which was sensitive to penicillin and ceftriaxone. *Mycobacterium tuberculosis* in the abscess fluid was ruled out with acid-fast bacilli, PCR and culture. Her antibiotic regimen was changed to mezlocillin/sulbactam 3.375 IVGTT q8h for 2 weeks. Following patient had major clinical improvement with temperature becoming normal and alleviated chest pain. She was discharged with a two-weeks oral amoxicillin-clavulanate course (0.375 mg tid). A total of 22 months post-discharge the patient was clinically stable. A repeat CT (Fig. 1c, d) and bronchoscopy showed complete resolution of the mediastinal abscesses and pneumonia. Meanwhile, the calcified mediastinal lymph node was reduced to 12 × 9 mm. The final follow-up was on January 22, 2019.

## Discussion

DNM usually originates from oral or neck infection in most cases, and often becomes fatal without surgical drainage if the infection and inflammation spread widely in the mediastinum [5]. It is associated with high mortality unless it is diagnosed and treated promptly. This disease has become generally uncommon since the introduction of antibiotic therapy decades ago [6]. Early surgical mediastinal drainage is strongly recommended for DNM [7].

The causative agent *Streptococcus constellatus* is generally anaerobic and most commonly known as Miller Streptococcus [8]. More recently, a growing list has surfaced of conditions identified as potential predisposing factors, including chest trauma, chest surgery, preexisting pericardial disease, uremia, collagen vascular disease, alcohol abuse, malignancy and immunosuppression [9]. It could be hypothesized that the giant calcified mediastinal lymph node causes a possible endobronchial leak. Following an inhalation of *Streptococcus constellatus* originated from the upper airways could develop descending necrotizing mediastinitis. Simultaneously, repetitive inflammation stimulated the formation of inflammatory granuloma. This benign lesion was treated with bronchoscopic biopsy forceps and never cured by an ablative endoscopic procedure such as laser therapy, argon plasma coagulation, cryoablation. It was disappeared in the following reexamination.

Searching the English medical literature in PubMed, we identified only three cases of necrotizing mediastinitis infection caused by *Streptococcus constellatus* from 2011 to 2017. The first case was reported by Oshima M and colleagues in 2011 [10], the second by Bhatt YM et al. in the same year [11] and the third by Kaiho T et al. in 2017 [12]. Therefore, the current report presented the fourth case in which *Streptococcus constellatus* infection caused mediastinal abscesses. Bhatt YM et al. [11] described a healthy 77-year-old man who presented with difficulty in swallowing saliva. Contrast-enhanced CT examination showed gas and fluid in the deep neck space. However, initial symptoms in this patient pointed mostly to a possible acquired pneumonia that was evidenced by clinical and radiologic findings. Early recognition of changes in both contrast-enhanced CT and Endobronchial Ultrasound-Guided Transbronchial Needle Aspiration (EBUS-TBNA) were key elements for timely diagnosis and immediate intervention. Oshima M et al. [10] presented a 61-year-old man who complained of neck swelling after tooth extraction; cervial drainage was performed for the diagnosis of cervical abscess. Thoracic drainage alone was not effective and thoracotomy was performed to open the mediastinal pleura. EBUS-TBNA was key element for timely diagnosis and immediate intervention; it is not only effective in diagnosing necrotizing mediastinitis, but also assists in prompt mediastinal abscess fluid evacuation and helps avoid thoracotomy, hence improving the chances of recovery and survival [13].

Twenty-two months post-discharge, the patient was clinically stable. Last repeat CT and bronchoscopy showed complete resolution of the mediastinal abscesses and pneumonia. In addition, the calcified mediastinal lymph node was decreased.

## Conclusions

Historically, DNM seems to be reported more often due to a possibly increasing list of risk factors such as oral or neck infections in most cases. The *Streptococcus milleri* group, including *Streptococcus constellatus*, can uncommonly cause mediastinal abscess. While cases of a giant calcified mediastinal lymph node and a proximal right main bronchial inflammatory granuloma remain rare, the medical staff should improve their understanding of the diagnosis and treatment of such diseases. Immediately EBUS-TBNA should be implemented to drain the mediastinal abscess. It can greatly reduce the probability of mediastinal surgery, reduce hospitalization time and cost. Furthermore, positive adjustment of treatment regimen according to drug sensitivity will contribute to the improvement of prognosis of the disease. However it should be carried out only in centres with large experience in endobronchial ultrasound guided needle aspiration techniques in order to reduce the possible risks, such as mediastinal infections and sepsis.

## Data Availability

The data set supporting the results of this article are included within the article.

## Abbreviations

AFB: Acid-fast bacilli
ANA: Antinuclear antibody
BALF: Bronchoalveolar lavage fluid
BP: Blood pressure
CRP: C-reactive protein
DNM: Descending necrotizing mediastinitis
EBUS-TBNA: Endobronchial Ultrasound-Guided Transbronchial Needle
ECG: Electrocardiogram
ESR: Erythrocyte sedimentation rate
HIV: Human immunodeficiency virus
WBC: White blood count
